# Quantitative Flow Cytometry to Understand Population Heterogeneity in Response to Changes in Substrate Availability in *Escherichia coli* and *Saccharomyces cerevisiae* Chemostats

**DOI:** 10.3389/fbioe.2019.00187

**Published:** 2019-08-05

**Authors:** Anna-Lena Heins, Ted Johanson, Shanshan Han, Luisa Lundin, Magnus Carlquist, Krist V. Gernaey, Søren J. Sørensen, Anna Eliasson Lantz

**Affiliations:** ^1^Department of Chemical and Biochemical Engineering, Technical University of Denmark, Lyngby, Denmark; ^2^Glycom A/S, Hørsholm, Denmark; ^3^Section of Microbiology, Department of Biology, University of Copenhagen, Copenhagen, Denmark; ^4^Division of Applied Microbiology, Department of Chemistry, Lund University, Lund, Sweden

**Keywords:** population heterogeneity, quantitative flow cytometry, glucose pulse, reporter strain, membrane robustness, flow cytometry

## Abstract

Microbial cells in bioprocesses are usually described with averaged parameters. But in fact, single cells within populations vary greatly in characteristics such as stress resistance, especially in response to carbon source gradients. Our aim was to introduce tools to quantify population heterogeneity in bioprocesses using a combination of reporter strains, flow cytometry, and easily comprehensible parameters. We calculated mean, mode, peak width, and coefficient of variance to describe distribution characteristics and temporal shifts in fluorescence intensity. The skewness and the slope of cumulative distribution function plots illustrated differences in distribution shape. These parameters are person-independent and precise. We demonstrated this by quantifying growth-related population heterogeneity of *Saccharomyces cerevisiae* and *Escherichia coli* reporter strains in steady-state of aerobic glucose-limited chemostat cultures at different dilution rates and in response to glucose pulses. Generally, slow-growing cells showed stronger responses to glucose excess than fast-growing cells. Cell robustness, measured as membrane integrity after exposure to freeze-thaw treatment, of fast-growing cells was strongly affected in subpopulations of low membrane robustness. Glucose pulses protected subpopulations of fast-growing but not slower-growing yeast cells against membrane damage. Our parameters could successfully describe population heterogeneity, thereby revealing physiological characteristics that might have been overlooked during traditional averaged analysis.

## Introduction

Optimization of industrial cultivation processes requires comprehensive analysis and understanding of host strain physiology throughout the cultivation. Nevertheless, analysis of microbial cultures is traditionally performed using averaged cell characteristics from samples containing millions of cells (Fernandes et al., 2011; Dusny and Schmid, 2015; Heins and Weuster-Botz, 2018). However, microbial cells exhibit intrinsic cell-to-cell variability that is influenced by the cultivation parameters but also in itself affects overall process performance (Müller et al., 2010; Delvigne et al., 2017; Lemoine et al., 2017). Additionally to this omnipresent intrinsic heterogeneity, that even arises in stable environments and can lead to e.g., metabolic specialization (Nikolic et al., 2017), heterogeneity originating from extrinsic sources can evolve. In particular, large-scale cultivations introduce phenotypic heterogeneity through gradients of dissolved oxygen, substrate, temperature, and pH, exposing the circulating microbial cells to rapid environmental changes (Lara et al., 2006; Wang et al., 2015). Apart from affecting the cells' metabolism, dynamic cultivation conditions impose metabolic stresses on cells and create inherent population heterogeneity (Carlquist et al., 2012; Ackermann, 2015). Consequently, averaged response values fail to describe the influence of different subpopulations and may even mask important characteristics of single cells in a bioprocess (Díaz et al., 2010; Fernandes et al., 2011; Tibayrenc et al., 2011; Gonzalez-Cabaleiro et al., 2017).

Population heterogeneity of single cells in bioprocesses is nowadays mostly assessed by flow cytometry which enables the analysis of thousands of cells per second. This technique acquires and records large data sets related to several different properties (as forward scatter, side scatter and fluorescence) per cell. However, apart from the use of mean and standard deviation, respectively, the coefficient of variance, flow cytometry data are generally presented as single-parameter histograms or bi-plots correlating two parameters (Attfield et al., 2001; Kacmar et al., 2004; Heins and Weuster-Botz, 2018). Evaluating these classical histograms and bi-plots together with mean fluorescence following different samples taken during a bioprocess, general traits of a population distribution like (dis-)appearance of subpopulations, shifts in fluorescence intensity as well as distinct differences in the shape of a population can be observed. However, this data treatment encourages subjective person-dependent interpretation of changes in the level of population heterogeneity as a different and biased focus might be put on changes that are expected to happen. Moreover, the comparison of fluorescence distributions of samples taken following a bioprocess or an environmental change is not straightforward and small shifts and differences are easily overlooked. Some algorithms for quantitative investigation of fluorescence distributions exist as e.g., flow Cytometric Histogram Image Comparison (flowCHIC), which has been shown to be applicable to interpret variations in microbial community structures (Koch et al., 2013). However, they require special software packages and are not easily adaptable to evaluate temporal changes in samples collected from a bioprocess.

Therefore, the aim of this study was to find simple and objective person-independent tools for quantitative description of shape and fluorescence intensity of population heterogeneity data collected with flow cytometry during industrial scale bioprocesses while still preserving all relevant information. These parameters should be easy to comprehend, especially for bioprocess engineers that are normally non-experts in analyzing flow cytometry data. We demonstrate applicability of the introduced parameters to evaluate population heterogeneity related to growth and membrane robustness of chemostat cultures of slow- and fast-growing *Escherichia coli* and *Saccharomyces cerevisiae* cells in steady state, respectively, after glucose perturbation. In this way, we investigated how microbial population heterogeneity is influenced by changes in nutrient availability simulating fluctuating substrate conditions in industrial scale cultures.

To visualize population heterogeneity, fluorescent reporter strains are nowadays extensively applied (Polizzi and Kontoravdi, 2015; Davis and Isberg, 2016). We used promoter-based growth reporter strains of the industrially relevant organisms *S. cerevisiae* (Carlquist et al., 2012) and *E. coli* (Han et al., 2013) expressing GFP and quantified the responses with single-cell resolution. GFP was integrated downstream of the promoters. The promoters are ribosomal promoters whose activity is considered to be growth-related (Regenberg et al., 2006). The *S. cerevisiae* reporter strain has additionally been used for analysis of cell membrane robustness (Carlquist et al., 2012) making it a dual reporter, and therefore an interesting tool for investigation of responses to changes in nutrient availability. To evaluate cell membrane robustness, it was shown to be a convenient method to expose cells to freeze-thaw treatment prior to flow cytometry analysis. In this way, an inverse correlation between GFP signal and membrane permeabilization could be established (Carlquist et al., 2012). In accordance with earlier studies, the membranes of glucose-grown, exponential-phase cells were particularly susceptible to freeze-thaw damage and hence less robust whereas the membranes of slower growing cells during growth on ethanol after diauxic shift were more resilient (Lewis et al., 1993; Hua et al., 2004; Brauer et al., 2008; Taymaz-Nikerel et al., 2011; Carlquist et al., 2012). The *E. coli* reporter was selected based on its ability to rapidly respond to changing environmental conditions, and combined with destabilized GFP to avoid a “memory effect” on gene expression due to slow maturation and degradation characteristics of the fluorescent protein (Han et al., 2013). Furthermore, it has been demonstrated that the behavior of both reporter systems depends on the operating mode of the bioreactor (Carlquist et al., 2012; Han et al., 2013).

Although stress responses to perturbation have been studied, little is known about how changing environments affect population heterogeneity or how different subpopulations contribute to the cultivation process (Müller et al., 2010). In addition, studies on the transient metabolic responses of microorganisms to rapid changes in nutrient availability have used averaged population data (Lara et al., 2006; Müller et al., 2010; Fernandes et al., 2011). Specifically, external perturbations can alter enzyme activity and metabolic flux, destabilize cell structures, affect chemical gradients, and alter the intracellular pH (Gasch et al., 2000; Kresnowati et al., 2006; Lara et al., 2006; Mashego et al., 2006; Zakrzewska et al., 2011). However, they might also facilitate cell adaption to novel conditions (Bylund et al., 1998; Enfors et al., 2001; Avery, 2006) as it can lead to higher stress tolerance, especially in slow-growing cells (Gasch and Werner-Washburne, 2002; Kresnowati et al., 2006; Lara et al., 2006; Mashego et al., 2006; Zakrzewska et al., 2011). The concomitant stress responses following the pulse are typically transient gene expression changes that lead to new steady-state expression levels (Schweder et al., 1999; Ronen and Botstein, 2006; Wu et al., 2006; Delvigne et al., 2009; Taymaz-Nikerel et al., 2011; Yosef and Regev, 2011) with a magnitude and duration corresponding with the duration and magnitude of the perturbation.

We hypothesize that by applying our tools in combination with growth reporter strains, we will be able to confirm as well as supplement what is known about population heterogeneity in response to glucose perturbations without applying time-consuming omics methods that are also not yet available on single cell level (Heins and Weuster-Botz, 2018).

## Materials and Methods

### Strains

The growth reporter strains *S. cerevisiae* FE440 (Carlquist et al., 2012) and *E. coli* MG1655/pGS20P*fis*GFPAAV (Han et al., 2013) were used in this study. FE440 stably expresses enhanced green fluorescent protein (GFP) from the ribosomal RPL22a promoter (Carlquist et al., 2012). MG1655/pGS20*fis*GFPAAV carries a low-copy plasmid that expresses unstable GFP from the *fis* promoter, which controls a transcriptional activator for the bacterial ribosomal promoter gene *rrnB*. The destabilized GFPAAV GFP variant achieves sufficiently high GFP fluorescence and permits the observation of rapid changes, which cannot be captured in yeast due to the longer generation time (Han et al., 2013). Plasmids were maintained in *E. coli* cultures with 25 μg mL^−1^ chloramphenicol, which did not interfere with normal growth behavior of the strain (data not shown).

### Cultivation Conditions

#### Pre-cultures

*S. cerevisiae*: Single colonies were used to inoculate 0.5 L baffled shake flasks with 100 mL defined mineral medium according to Verduyn et al. (1992) and 10 g L^−1^ glucose. Pre-cultures were incubated with shaking at 150 rpm and 30°C to mid-exponential phase (~6 h).

*E. coli*: Single colonies were used to inoculate 100 mL Luria Bertani broth in shake flasks for growth overnight at 37°C. Serial dilutions in 10-fold steps were afterwards incubated at 37°C for 6 h with shaking at 180 rpm. Cultures with optical density (OD_600_) values of 0.4–0.6 were used to inoculate bioreactors.

#### Chemostats

*S. cerevisiae:* Aerobic, glucose-limited, level-based chemostat cultures were grown with two dilution rates, D = 0.05 h^−1^ and 0.30 h^−1^, in biological triplicates in 1 L bioreactors with 0.8 L working volume (Sartorius, B. Braun Biotech International GmbH, Melsungen, Germany). Probes for oxygen tension and pH (Mettler Toledo, OH, USA) were calibrated according to procedures provided by the manufacturer using two-point calibration (pH 4 and 7, gassing with oxygen and nitrogen, respectively). Defined mineral medium according to Verduyn et al. (1992) was used with 5 g L^−1^ glucose. OD_600_ at inoculation was 0.001. The pH was held at 5.0 using 2 M NaOH. Cultivation parameters were kept constant: 30°C, 1 vvm aeration and 600 rpm.

*E. coli:* Glucose-limited triplicate level-based chemostat cultures were inoculated with OD_600_ 0.01 and operated at 37°C, 1 vvm and 1,000 rpm in the same reactors as for yeast, but with a 0.6 L working volume. Defined mineral medium according to Xu et al. (1999) with 4.5 g L^−1^ glucose was used.

The batch phase of yeast and *E. coli* was followed by measurement of OD_600_, determination of dry cell weight and mass spectrometry for off-gas composition (Prima Pro Process MS, Thermo Fisher Scientific, Winsford UK). After glucose depletion, detected as a rapid drop in CO_2_, cultures were switched to chemostat mode applying a medium feed with the same composition as for the batch at flow rates to achieve the indicated D. Steady state was established when dry weight, dissolved oxygen tension and exhaust CO_2_ were constant for at least three residence times. After confirming steady state, cultures were perturbed by addition of a concentrated glucose solution with a final bioreactor concentration of 1 g L^−1^ for *S. cerevisiae* and 0.45 g L^−1^ for *E. coli*, with subsequent frequent sampling depending on the organism.

Samples for OD_600_ and dry cell weight were analyzed directly. High performance liquid chromatography (HPLC) samples were sterile filtered and stored at −20°C. Flow cytometry samples were mixed with 15% glycerol and stored at −80°C. Yeast flow cytometry was also performed on non-frozen samples for D = 0.05 and 0.3 h^−1^; broth was mixed with 15% glycerol and kept on ice.

### Sample Analysis

#### OD_600_, Dry Cell Weight, and HPLC

Growth was monitored as OD_600_ with a spectrophotometer (UV mini 1240 spectrophotometer, Shimadzu, Kyoto, Japan). Dry cell weight was measured from 5 mL culture according to Olssen et al. (1998). Concentrations of glucose, acetate, ethanol, glycerol, and pyruvate in *S. cerevisiae* samples were determined by HPLC as described previously (Carlquist et al., 2012). *E. coli* cultures were additionally analyzed for lactate and formate. Glucose concentration was also measured by a hexokinase colorimetric procedure (ABX Pentra Glucose HK CP).

#### Flow Cytometry

*Sample preparation:* Non-frozen samples were kept on ice and frozen samples were thawed on ice before centrifuging for 1 min at 3,000 × g, 4°C and re-suspended in 0.9% saline solution for analysis. *E. coli* cells used for investigation of pH effects on GFP fluorescence were re-suspended in 100 mM phosphate buffer at pH 5.5 or 7, incubated 20 min at room temperature and kept on ice until analysis.

*Single-cell analysis:* Single-cell analysis used a BD FACS Aria III (Becton Dickinson, NJ, USA) with a 20 mW argon ion laser of 488 nm (focused to an elliptical spot 9 +/−3 μm high 65 +/−7 μm wide, Becton Dickinson, NJ, USA) for excitation. Fluorescence was collected with two scattering channels, forward scatter (FSC, photodiode with 488 nm/10 bandpass filter) and side scatter (SSC, photomultiplier with 488/10 nm bandpass filter), and one fluorescence detection channel of 530/30 nm (photomultiplier, bandpass filter) in the flow cytometer. The flow cytometer optics were aligned using 2.5 μm fluorescent polystyrene beads (AlignFlowTM, Thermo Fisher, MA, USA). Photo-multiplying tubes logarithmic amplification voltages were set based on negative and positive controls (yeast: FSC−366 V, SSC−375 V, 530/30 nm−535 V; *E. coli*: FSC−310 V, SSC−316 V, 530/30 nm−520 V). For yeast experiments, 20,000 events were recorded with a rate of ~1,000 events per seconds. For *E. coli* experiments, 30,000 events were recorded at the same speed. Cytometer Setup and Tracking beads (CS&T Research Beads, BD™, Becton Dickinson, USA) were used for automated quality assurance and control of machine performance. During measurement, collected fluorescence data were visualized in the flow cytometer built-in software BD FACSDivaTM v 6.x.

### Data Analysis

Raw flow cytometry data (fcs files) were loaded into MATLAB® R2017b (The MathWorks, Inc., Natick, MA, USA) using the readfcs function (by L. Balkay, University of Debrecen, Hungary, available on MATLAB® central file sharing). The readfcs function reads standard flow cytometry FCS format data. GFP fluorescence data were extracted from the fcs files as matrix containing both parameters as row vectors and saved into mat files. The empirical cumulative distribution function (cdf) from 1,024 recording channels of the flow cytometer was fitted to GFP fluorescence data using the MATLAB® built-in cdfplot-function.

The MATLAB® built-in hist-function creates a histogram bar chart of GFP fluorescence vectors by sorting its elements into 1,024 uniformly sized bins equal to the recording channels of the flow cytometer. This enabled plots of channel number (chrn) fluorescence, respectively, fluorescence intensity for the GFP detector as relative cell count per recording channel. To quantify the shape and intensity of GFP distributions, peak width at baseline level in 2D histogram plots, defining over how many channel numbers the population distribution spreads, was calculated by first searching for the channel numbers in which at least 5 cell counts were registered. The number of cell counts was defined based on the number of random cell counts in channel numbers outside the population distribution that was found to be 2 ± 1. In this way, a clear identification of distributions was possible. The peak width was then determined by subtracting the lowest channel number with more than 5 cell counts from the highest channel number in which more than 5 cell counts were registered.

The MATLAB® built-in mean function, that returns the average of a fluorescence vector, was used to calculate mean FSC and GFP fluorescence. Normalized GFP fluorescence was estimated by dividing mean GFP fluorescence by OD_600_.

The coefficient of variance (CV) value, which is also a measure of noise in gene expression (Silander et al., 2012), was generated by dividing peak width by mean GFP. Cdfplot slopes were estimated by fitting a line to the inflection point of the cumulative distribution of GFP fluorescence histograms using the MATLAB® built-in polyfit-function with a degree of one. The polyfit-function returns the coefficients for a fitted polynomial function with a specified degree that is a best fit according to the least-squares method for the available fluorescence data. The skewness of a population distribution was determined through the relationship between mode and mean of a distribution. The MATLAB® built-in mode-function was applied to determine the mode of fluorescence vectors, which was then subtracted from the mean, returning positive values for right skew, respectively, negative values for left skew distributions.

For frozen, glucose-pulsed samples, the percentage of cells that form two subpopulations, where one sub-population was developing during freeze-thaw treatment, was computed as the ratio between the cell numbers in the subpopulations and the total cell number collected in the sample. For that purpose, histogram plots where divided into three channel number ranges of low, middle, respectively, high fluorescence intensity set by their relation to the local minimum between two subpopulations. The local minimum between the subpopulations was determined using the MATLAB® built-in min-function that returns the minimum cell count in a specified range of channel numbers. The middle fluorescence range was defined as the local minimum ±5% fluorescence and excluded to disregard potential overlap of the two subpopulations. High-range and low-range subpopulation percentages were calculated by dividing cell numbers in the respective ranges by the total cell number recorded. All values and estimated parameters are derived from biological triplicates, which is indicated by vertical error bars.

## Results

Investigation of population heterogeneity in bioprocesses employing flow cytometry analysis often results in multiple fluorescence distribution data for e.g., a fluorescence marker and FSC collected from consecutive samples following the process or responses to environmental changes that have to be quantitatively compared in shape and fluorescence intensity (Heins and Weuster-Botz, 2018). Mean fluorescence is already widely used to describe the general trends of an averaged fluorescence distribution (Fernandes et al., 2011). As a stand-alone variable, mean fluorescence leads to significant loss of information compared to interpretation of raw distribution data. Therefore, we apply additional parameters that can be used in combination with mean fluorescence to quantitatively describe population heterogeneity in bioprocesses presented in histogram plots, without losing details. Some of these parameters describe similar characteristics of fluorescence distributions which enables us to give recommendations for the best combination of parameters.

The coefficient of variance (CV), corresponding to the ratio between the width of a distribution and the mean fluorescence, can provide information about the level of heterogeneity of a population. However, it also provides information about noise in gene expression if the fluorescence that forms the basis for the distribution is related to expression of a specific gene (Silander et al., 2012). Low values describe a narrow distribution with uniform cells, respectively, low levels of noise, whereas high values point toward higher noise levels and a broader distribution, potentially also appearance of subpopulations and therefore a higher level of heterogeneity. Peak width at baseline level in histogram plots defines over how many channel numbers a fluorescence distribution spreads, and this variable is also higher for more heterogeneous populations. Furthermore, the mode, meaning the channel number with the highest cell count of the histogram, which can, depending on the shape of the fluorescence distribution be significantly different from the mean, is introduced. The mode can be used to calculate the skewness of a population distribution by calculating the difference between mean and mode. When distributions show a right skew the skewness is positive whereas negative values are found for left skew distributions. Clearer skewness is indicated by higher values for the skew. Normal distributions do not exhibit a skew, and consequently the skewness is zero. Hence, skewness can give information about temporal shifts in fluorescence intensity, the distribution shape or potential subpopulations adjacent to the main population. Alternatively, the slope at the inflection point of the cumulative distribution function (cdf) curve can be used, which can highlight changes in distribution shape and the appearance of potential subpopulations when the slope is significantly decreasing between consecutive samples. Generally, broad distributions exhibit low slope values whereas high slope values are found for narrow distributions. Additionally, if significant subpopulations appear, the subpopulation percentage can be calculated, which is defined as the ratio between cell numbers in subpopulations and total cell number collected in the sample. It has to be noted that the term subpopulation means the appearance of a bimodal distribution. The different subpopulations can however be adjacent to each other in the histogram.

To demonstrate the applicability of the combination of these parameters for interpretation of flow cytometry data collected in bioprocesses, we investigated the influence of growth rate and glucose excess on microbial population heterogeneity assessed through GFP fluorescence expressed from ribosomal-related promoters, and cell membrane robustness, measured as response to freeze-thaw treatment. After characterization of steady state, glucose pulses were introduced to glucose-limited continuous cultures of *S. cerevisiae* reporter strain FE440 (Carlquist et al., 2012) and *E. coli* reporter strain MG1655/pGS20P*fis*GFPAAV (Han et al., 2013). Cultivations with both, low and high dilution rate, were performed to compare responses of cells in a fully respiratory vs. a respiro-fermentative growth state. High D (0.3 h^−1^ for yeast and 0.51 h^−1^ for *E. coli*) corresponded to about 77% of the maximum specific growth rate, and low D (0.05 h^−1^ for yeast and 0.1 h^−1^ for *E. coli*) to ~13% of the maximum specific growth rate (Larsson et al., 1993; Nahku et al., 2010). An additional D = 0.36 h^−1^ experiment was used for *E. coli* to also study conditions that are in the vicinity of the dilution rate at which overflow metabolism occurs (Nanchen et al., 2006).

### Population Growth Characteristics of Steady-State Yeast Cultures

Distributions of GFP fluorescence and cell size parameters as FSC at different dilution rates in steady state were investigated using flow cytometry on freshly harvested cells (referred to as fresh cells). For a dilution rate of 0.05 h^−1^, cells grew, consistent with earlier studies (Postma et al., 1989; Hoek et al., 1998; Diderich et al., 1999; Dijken et al., 2000), fully respiratory without overflow metabolism, whereas at D = 0.3 h^−1^ respiro-fermentative metabolism was found (see Supplementary Material S1 and Table S1).

**Figure 1:**
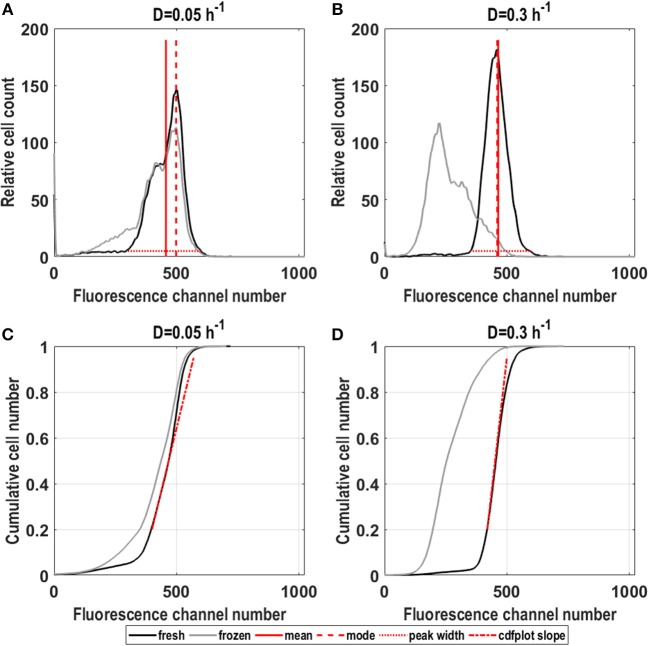
Fluorescence distributions for fresh and frozen *S. cerevisiae* FE440 cells in steady-state, aerobic glucose-limited chemostats. Cell count against fluorescence channel number for fresh and frozen cells grown at D = 0.05 h^−1^ **(A,C)** and D = 0.3 h^−1^ **(B,D)** and cdfplots of cumulative cell number against fluorescence channel number **(C,D)** for both dilution rates for fresh and frozen cells. Red lines indicate mean (full line), mode (dashed line), peak width (dotted line), and cdfplot slopes (dash-dotted line) calculated for the depicted distributions. All plots show average values from three different residence times originating from biological triplicates.

Histogram plots for GFP fluorescence commonly used for analysis of flow cytometry data (Fernandes et al., 2011; Gonzalez-Cabaleiro et al., 2017), are presented in Figure 1. GFP fluorescence distributions (Figures 1A,B) for D = 0.05 h^−1^ showed cells grouped into a main population and a subpopulation of lower fluorescence adjacent to the main population. The main high fluorescent subpopulation covered a broader fluorescence range in the 0.05 h^−1^ than in the 0.3 h^−1^ cultures (Figures 1A,B). Using the combination of parameters, the analysis can be extended. Growth related GFP fluorescence distributions for cells grown at high D were more narrow and distinct than distributions for cells grown at low D (Figures 1A,B, fresh cells), which is also described by higher peak width values, lower CV and the lower cdf plot slope for D = 0.05 h^−1^ (Table 1 fresh cells and depicted in Figures 1C,D red dotted line). However, mean GFP fluorescence did not significantly differ for both dilution rates so that lower D cultures only showed slightly more distribution variation (higher CV), indicating a higher degree of population heterogeneity (Table 1). When correlating CV to noise in gene expression, the inverse correlation between gene expression and noise in gene expression found in an earlier study (Baert et al., 2015) was also confirmed here.

**Table 1:** Quantification of yeast growth related population heterogeneity in steady-state reached for dilution rates of D = 0.05 h^−1^ and D = 0.3 h^−1^.

**Parameter^a^**		**D = 0.05 h^**−1**^**	**D = 0.3 h^**−1**^**
Mean GFP	Fresh	456.9 ± 28.3	464.6 ± 20.3
	Frozen	409.4 ± 14.3	295.7 ± 6.9
CV GFP	Fresh	0.18	0.16
	Frozen	0.26	0.28
Peak width	Fresh	271 ± 15.6	227 ± 42.4
	Frozen	551 ± 7.2	381 ± 6.5
Cdf plot slope	Fresh	0.0062 ± 2.2·10^−4^	0.0095 ± 5.2·10^−4^
	Frozen	0.0060 ± 1.2·10^−4^	0.0061 ± 7.1·10^−4^
Mode GFP	Fresh	498.5 ± 4.9	460.5 ± 15.8
	Frozen	486.5 ± 17.7	230.0 ± 29.7
Skewness GFP	Fresh	−26.9 ± 6.5	4.4 ± 1.2
	Frozen	−78.8 ± 2.4	54.3 ± 16.1

a *Standard deviations obtained from biological triplicates*.

Through the difference between mean and mode (Figures 1C,D depicted as red line, respectively, red dashed line and Table 1), which is for the 0.05 h^−1^ cultivation larger than for 0.3 h^−1^, the skewness can be calculated. Distributions for the 0.05 h^−1^ cultivation exhibit a strong left skew whereas only a slight right skew is detected for the higher dilution rate.

### Membrane Robustness of Yeast Cells in Steady-State

Robustness of *S. cerevisiae* in steady state was investigated using the reporter strain to identify fluorescence responses to freeze-thaw treatment, with the GFP signal as a measure of membrane integrity. This method was established in an earlier study (Carlquist et al., 2012). Preliminary experiments (see Supplementary Material S2) revealed, that GFP fluorescence decrease in yeast cells with permeabilized membrane after freezing is likely connected to reduced intracellular pH. We applied the combination of parameters to quantitatively evaluate changes in distribution shapes compared to fresh cells and how growth rate affected membrane integrity in frozen samples from D = 0.05 and 0.3 h^−1^ cultures. D affected the response to freeze-thaw treatment (Figures 1A–D). For D = 0.05 h^−1^, freezing did not change the distribution shape of the main population, manifested in similar cdfplot slopes and a fairly constant mode of the fluorescence (Figure 1C, Table 1). However, due to a slight increase in the lower fluorescence population, the CV and peak width increased by about 31%, respectively, 51% together with a decrease in mean fluorescence and a significant increase in left-sided skewness.

In contrast, for D = 0.3 h^−1^, mean fluorescence decreased by around 36% and the distribution broadened (Table 1). Consequently, the CV increased and the cdfplot slope for frozen cells was considerably lower than for fresh cells (Figure 1D, Table 1). Furthermore, the distributions showed broad tailing toward higher fluorescence, which is manifested in an about 91% increase in right-sided skewness, and a division into two adjacent distinct populations as apparent consequences of freeze-thaw treatment (Figure 1B, Table 1). These results suggest that cells growing with a lower growth rate are more robust toward freeze-thaw treatment, which might be connected to redistribution of cellular resources to serve survival and growth when nutrients are scarce (Carlquist et al., 2012).

### Influence of Glucose Perturbation on *S. cerevisiae* Population Heterogeneity

After description of the differences between single distributions, we evaluated whether our parameters can also be employed to describe timely changes in distribution shape and fluorescence intensity. For that purpose we investigated population heterogeneity in response to a concentrated glucose pulse of 1 g.L^−1^.

**Figure 2:**
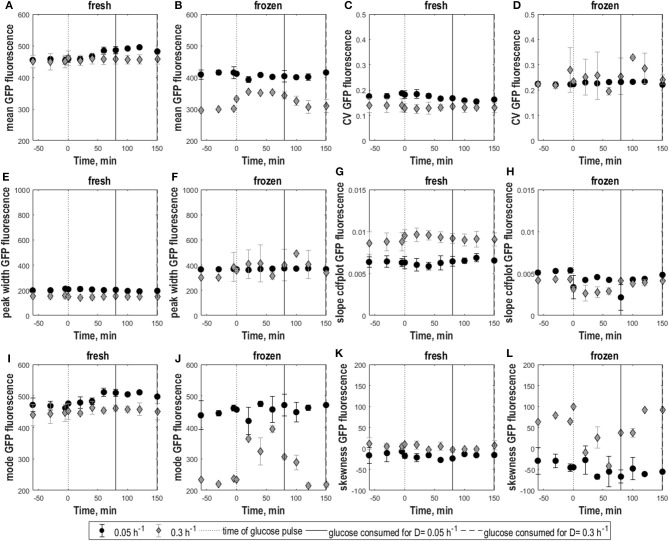
Population heterogeneity of fresh and frozen yeast cells following glucose perturbation. Mean GFP fluorescence **(A,B)**, CV of GFP fluorescence **(C,D)**, peak width **(E,F)** and cdfplot slope **(G,H)**, mode of GFP fluorescence **(I,J)**, skewness of GFP fluorescence **(K,L)** vs. time following a 1 g.L^−1^ glucose pulse for *S. cerevisiae* FE440 in aerobic, glucose-limited chemostat cultures at D = 0.05 h^−1^ (circles) and D = 0.3 h^−1^ (diamonds) for fresh **(A,C,E,G,I,K)** and frozen cells **(B,D,F,H,J,L)**. The black dotted line indicates the time of the glucose pulse. Full, respectively, dotted vertical lines indicate the time when pulsed glucose was consumed under the indicated culture conditions. Vertical error bars indicate biological triplicates.

Whereas low D cultures (0.05 h^−1^) displayed batch-culture-like behavior with overflow metabolism when perturbed, consistent with (Visser et al., 2004), respiro-fermentative cultures (0.3 h^−1^) consumed the extra glucose at a slower rate than slower-growing cultures and did not show increased ethanol production (see Supplementary Material S3 and Figure S1). Consistently, the glucose pulse caused a slight upshift in GFP fluorescence for non-frozen cells grown at 0.05 h^−1^ reflected in increasing mean and mode fluorescence until 30–40 min after the glucose pulse. In contrast, fluorescence of cells grown at 0.3 h^−1^ remained unchanged with constant mean and mode fluorescence (Figures 2A,I,K fresh). Consistently, skewness remained constant, revealing that the shape of distributions collected for both dilution rates remained the same as in steady-state. Consistently, as in steady-state CV and peak width (Figures 2C,E fresh) indicated somewhat higher heterogeneity for cultures with low growth rate with no impact from the glucose pulse. Accordingly, the cdfplot slope (Figure 2G) increased after glucose perturbation for D = 0.3 h^−1^ and decreased for D = 0.05 h^−1^, because of a decline in the low fluorescence subpopulation for the D = 0.3 h^−1^ culture, respectively, an increase in this subpopulation for the D = 0.05 h^−1^ culture. When the pulsed glucose was depleted, slope values returned to steady-state values.

### *S. cerevisiae* Cell Robustness After Glucose Perturbation

Membrane integrity of cells grown at D = 0.05 h^−1^ and D = 0.3 h^−1^ was differently affected by glucose pulses. Frozen cells from 0.05 h^−1^ cultures had similar GFP fluorescence distributions before and after the glucose pulse with constant mean fluorescence, indicating that membrane integrity was not affected (Figure 2B). However, the broader population distribution of frozen cells at low D got more pronounced after the glucose pulse, as seen by higher CV and peak width compared to fresh cells as well as a decrease in cdfplot slope and increase in skewness in response to the pulse (Figures 2D,F,H,J,L).

**Figure 3:**
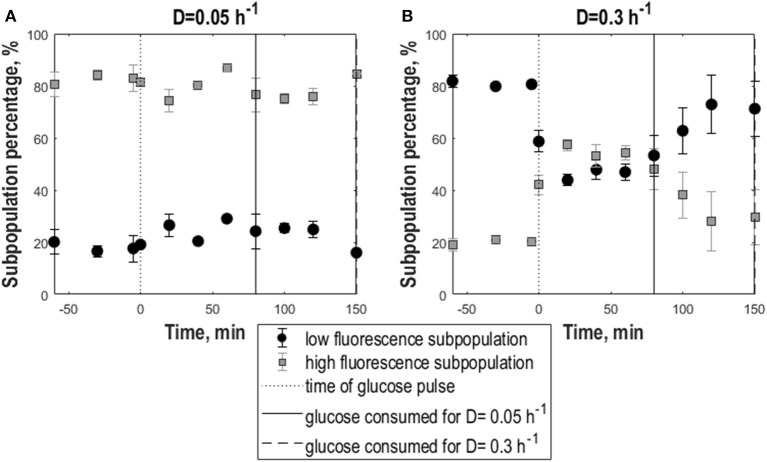
Subpopulation distribution dynamics following glucose perturbation for yeast cells as a consequences to exposure to freeze-thaw treatment. Percentage of low-fluorescence (circles, black) and high-fluorescence (squares, gray) subpopulations vs. time following a 1 g.L^−1^ glucose pulse of *S. cerevisiae* FE440 in aerobic, glucose-limited chemostat cultures frozen after growth at D = 0.05 h^−1^ **(A)** or D = 0.3 h^−1^ **(B)**. The black dotted line indicates the time of the glucose pulse. Full, respectively, dotted vertical lines correspond to the time when pulsed glucose was consumed for the cultures grown at D = 0.3 h^−1^ (dotted vertical line) and D = 0.05 h^−1^ (full vertical line). Vertical error bars indicate biological triplicates.

In contrast, frozen cells from 0.3 h^−1^ cultures showed an increase in the percentage of high-fluorescence population in response to the pulse reflected as an increase in GFP fluorescence mean and mode (Figures 2B,J). Additionally, the distributions slightly widened resulting in a decrease of the cdfplot slope and at the same time an increase in CV, peak width and right-sided skewness (Figures 2D,F,H,L). To further quantify the effect of the glucose pulse on the changes in subpopulation distribution after freeze-thaw treatment, additionally the relative fractions of low and high-fluorescence subpopulations were calculated for different time points (Figure 3). In steady state, the low-fluorescence subpopulation fraction of high D cultures comprised 80% of the population. When glucose was added, this changed to an approximately equal distribution. For 0.05 h^−1^ cultures, the glucose pulse had no effect on the changes in subpopulation distribution after freeze-thaw treatment with approximately 20% low-fluorescence cells. Although cells grown at 0.3 h^−1^ were more affected by freezing than cells grown at low D, glucose appeared to protect fast-growing cells against freeze-thaw treatment.

### Population Characteristics of Steady-State *E. coli* Cultures

After demonstrating that our tools for quantitative analysis of population heterogeneity can be used for yeast cultures, we tested their applicability for bacteria with a culture of the *E. coli* growth-reporter strain to measure membrane robustness after freeze-thaw treatment. Preliminary experiments were conducted to determine if freezing resulted in leaky membranes (see Supplementary Material S4 and Figure S2). Cell samples were frozen and analyzed in buffers of pH 5–7.5 while ensuring that no degradation or synthesis of the fluorescence protein occurred. Since GFP fluorescence declined in buffers with a pH value below 6, we analyzed the membrane robustness of *E. coli* using GFP fluorescence measurements of frozen cells at pH 5.5 and 7, with GFP fluorescence at pH 7 as reference for intact cells.

**Figure 4:**
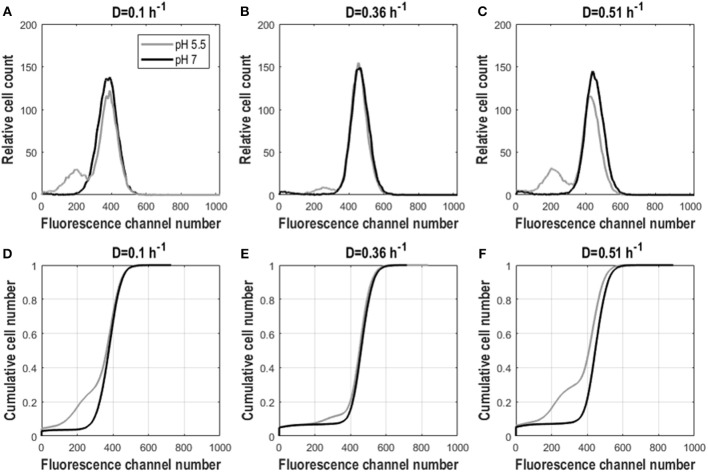
Fluorescence distributions for frozen *E. coli* cells in steady-state aerobic glucose-limited chemostats analyzed at pH 5.5 and 7. Cell count against fluorescence channel number **(A–C)** and cdfplots of cumulative cell number against fluorescence channel number **(D–F)** for D = 0.1 h^−1^, D = 0.36 h^−1^, and D = 0.51 h^−1^ for frozen *E. coli* cells analyzed at pH 5.5 (gray) and 7 (black). Plots show averaged values from three different residence times originating from biological triplicates.

As for yeast, we investigated the effect of growth rate on *E. coli* GFP expression and population heterogeneity. For dilution rates 0.1 and 0.36 h^−1^, cells grew fully respiratory without acetate formation, whereas above 0.4 h^−1^ respiro-fermentative metabolism was found, with a lower growth rate than reported in Kayser et al. (2005) and Nanchen et al. (2006), but consistent with (Hua et al., 2004) (see Supplementary Material S5 and Table S2). Cultures with different D had similar population distributions for GFP fluorescence but showed substantial differences when analyzed at pH 5.5 and 7. Intact cells (analyzed at pH 7) exhibited a single fluorescent population with a narrow distribution and therefore low CV and peak width (Table 2). When cells were analyzed at pH 5.5 a population with lowered fluorescence appeared, which is indicative for lowered membrane integrity, in addition to the main population of intact cells. This was manifested by a decreased mean as well as an increased mode, CV and peak width, compared to cells analyzed at pH 7 (Figures 4D–F and Table 2). Additionally, due to appearance of the population with lower fluorescence when cells were analyzed at pH 5.5, distributions exhibited a slight left-sided skew whereas for cells analyzed at pH 7 the opposite was the case. The low-fluorescence population appearing during analysis at pH 5.5 was more pronounced for D = 0.1 h^−1^ and D = 0.51 h^−1^ than for the intermediate dilution rate (Figures 4A–C). Accordingly, mean fluorescence remained highest for D = 0.36 h^−1^ for cells analyzed at pH 5.5. Cdfplot slopes (Table 2) were unaffected by analysis pH, confirming that only a small fraction of cells changed membrane integrity, whereas for the majority of cells it remained the same (Figures 4D–F). These results suggest that while GFP fluorescence increases with growth rate, the cell robustness decreases with growth rate, especially during respiro-fermentative metabolism.

**Table 2:** Quantification of *E. coli* population heterogeneity in steady-state reached for dilution rates of D = 0.1 h^−1^, 0.36 h^−1^, and 0.51 h^−1^, where frozen cells were analyzed at pH 5.5 and 7.

**Parameters^a^**	**pH**	**D = 0.1 h^**−1**^**	**D = 0.36 h^**−1**^**	**D = 0.51 h^**−1**^**
Mean GFP	7	377.5 ± 17.3	438.4 ± 4.2	422.5 ± 7.1
	5.5	359.9 ± 19.3	407.8 ± 23.2	370.7 ± 14.5
CV GFP	7	0.24	0.28	0.29
	5.5	0.32	0.35	0.39
Peak width	7	262.2 ± 12.5	232.7 ± 4.5	230.3 ± 5.1
	5.5	402.7 ± 9.4	419.7 ± 17.9	444.0 ± 2.7
Cdf plot slope	7	0.006 ± 4.8·10^−4^	0.007 ± 6.0·10^−4^	0.006 ± 3.3·10^−4^
	5.5	0.007 ± 2.5·10^−4^	0.007 ± 1.8·10^−4^	0.007 ± 5.7·10^−5^
Mode GFP	7	370.3 ± 13.5	425.7 ± 4.8	415.4 ± 6.3
	5.5	385.4 ± 19.3	411.3 ± 20.7	389.1 ± 15.1
Skewness GFP	7	9.1 ± 2.7	12.4 ± 0.4	7.5 ± 0.6
	5.5	−5.0 ± 0.7	−2.3 ± 1.8	−18.7 ± 0.4

a *Standard deviations obtained from biological triplicates*.

### Influence of Glucose Perturbation on *E. coli* Single Cell Physiology

**Figure 5:**
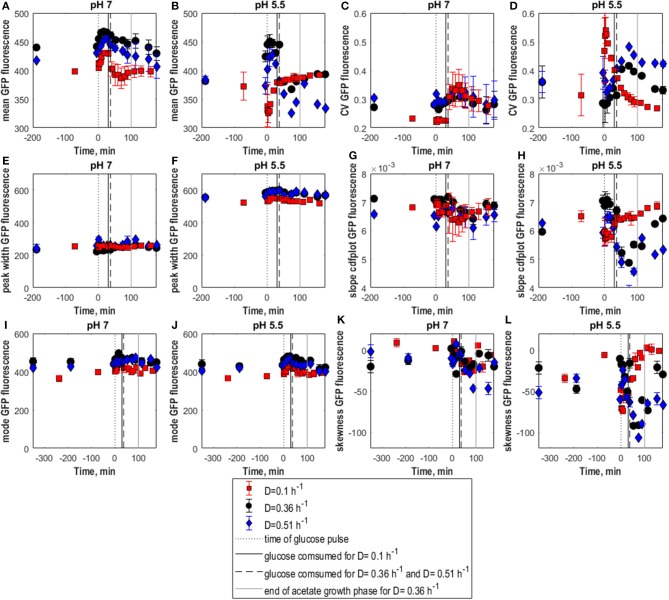
Population heterogeneity of *E. coli* following glucose perturbation analyzed at pH 5.5 and 7. Mean GFP fluorescence **(A,B)**, CV of GFP fluorescence **(C,D)**, peak width **(E,F)**, and cdfplot slope **(G,H)**, mode of GFP fluorescence **(I,J)**, skewness of GFP fluorescence **(K,L)** vs. time following a 0.45 g.L^−1^ glucose pulse for *E. coli* in aerobic glucose-limited chemostat cultures at D = 0.1 h^−1^ (squares), 0.36 h^−1^ (circles), 0.51 h^−1^ (diamonds) for cells analyzed at pH 7 **(A,C,E,G,I,K)** and 5.5 **(B,D,F,H,J,L)**. Black dotted line, time of glucose pulse. Full, respectively, dashed vertical lines, correspond to the time when pulsed glucose was consumed under the indicated culture conditions. A gray vertical line indicates the end of the acetate growth phase. Vertical error bars indicate biological triplicates.

Also for *E. coli* timely changes in distribution shape and fluorescence intensity were quantified following glucose pulses of 0.45 g.L^−1^ introduced to steady-state cultures. Pulsed glucose was depleted after 30 min for D = 0.1 h^−1^ and after 37 min for D = 0.36 h^−1^ and D = 0.51 h^−1^. Consistent with earlier studies for respiratory metabolism, no formation of acetate or other metabolites was detected as a consequence of the pulse at D = 0.1 h^−1^ (Kayser et al., 2005). For D = 0.36 h^−1^ and D = 0.51 h^−1^ overflow metabolism with subsequent consumption of acetate was found in response to the pulse (see Supplementary Material S6 and Figure S3 for data). Considering GFP fluorescence for intact cells (analyzed at pH 7), glucose pulses caused an upshift in mean fluorescence for all three D values (Figure 5A). With glucose depletion, fluorescence returned with an increase in population variance (CV and cdfplot slope, Figures 5C,G) to steady-state levels for D = 0.1 h^−1^ while for the two higher D values fluorescence decreased but remained higher than steady-state levels. This might be connected to acetate growth which was accompanied by a decrease in slope (Figure 5G). Possibly, the cells used noise in gene expression while growing on acetate, as also found earlier (Heins et al., 2019). Consequently, the reporter strain could capture both phases, however, with a less dynamic response for the lowest D. For D = 0.1 h^−1^ only a slight decrease in slope was seen after glucose depletion. Generally, no increase in population segregation was found considering peak width. However, the peak shape changed as depicted by the increase in left-sided skewness (Figures 5E,K).

### *E. coli* Cell Robustness After Glucose Perturbation

**Figure 6:**
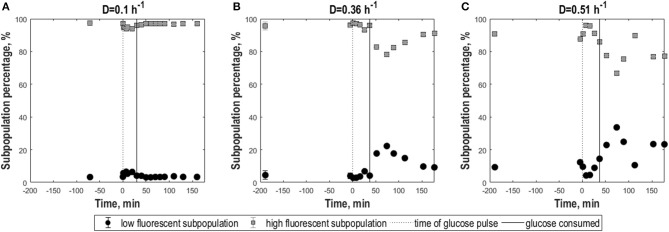
Subpopulation distribution dynamics following glucose perturbation for *E. coli* cells as a consequence of exposure to freeze-thaw treatment. Percentages of low-fluorescence (circles, black) and high-fluorescence (squares, gray) subpopulations vs. time following a 0.45 g.L^−1^ glucose pulse for *E. coli* in aerobic, glucose-limited chemostats cultures frozen after growth at D = 0.1 h^−1^ **(A)**, D = 0.36 h^−1^ **(B)**, and D = 0.51 h^−1^ **(C)**, measured at pH 5.5. The black dotted line indicates the time of the glucose pulse. Full vertical lines correspond to the time when pulsed glucose was consumed under the indicated conditions. Vertical error bars indicate biological triplicates, however errors were mostly insignificantly small.

When GFP fluorescence was analyzed at pH 5.5 to assess membrane robustness, slow-growing and fast-growing *E. coli* cells showed clear differences (Figure 5). As for yeast, complementary to the new parameters the relative fractions of low- and high-fluorescence populations appearing as a consequence of the analysis pH at different time points were calculated (Figure 6). For the two higher D values, membrane integrity changed depending on nutrient availability, with the mean GFP signal (Figure 5B) mirroring the overall trend of the CO_2_ evolution corresponding to glucose growth and acetate reassimilation. For D = 0.36 h^−1^, the low-fluorescence population with damaged membranes appearing at pH 5.5 compromised 5% in steady-state and increased to 10% with glucose perturbation. However, for D = 0.51 h^−1^, after an initial rise the fraction of damaged cells decreased by 5% with glucose pulse compared to steady state (Figures 6B,C). This was accompanied by a decrease in the level of heterogeneity, which can be observed by a slight slope increase and decrease in CV (Figures 5D,H). During growth on acetate after glucose depletion, membrane integrity was strongly affected as heterogeneity rose above steady state levels, which was manifested in a decrease in mean, a rise in CV and a drop in cdfplot slope (Figures 5B,D,H). Furthermore, the percentage of the damaged population fraction appearing at pH 5.5 increased further to 22%, respectively, 20%, which can also be observed by an increase in left-sided skewness. For D = 0.36 h^−1^, cultures slowly returned to steady state values after acetate depletion. For cultures at the highest D, the steady-state level was not reached again during the analysis period. For low D, membrane integrity was less affected; mean fluorescence, mode and left-sided skewness and cdfplot slope remained constant except for a slight rise, respectively, decrease during growth on the pulsed glucose (Figures 5B,H). Consistently, the low-fluorescence population appearing during analysis at pH 5.5 covered 3% prior to perturbation, which increased to 7% with the glucose pulse and returned to steady-state values with glucose depletion (Figure 6A). As a consequence of almost constant peak width, the CV at D = 0.1 h^−1^ showed a steep increase after glucose perturbation followed by an asymptotic decrease during glucose consumption (Figures 5D,F). These results suggest that the higher the growth rate, the more the cells are affected in robustness after the pulse, especially during the acetate consumption phase.

## Discussion

Analysis of population heterogeneity in bioprocesses is nowadays still mostly done on averaged values from single cell measurements or by person-dependent interpretation of bi-plots or single-parameter histogram plots (Fernandes et al., 2011; Heins and Weuster-Botz, 2018). To improve quantitative description of flow cytometry data, we described the shape and fluorescence intensity of single cell distributions, in addition to calculation of the mean fluorescence, using easily comprehensible parameters. We successfully applied our combination of parameters to measure cell-to-cell variability in growth and membrane robustness in *S. cerevisiae* and *E. coli* continuous cultures. Using reporter strains of both species, we quantified the effects of growth rate and a sudden change in glucose concentration on population variability mimicking the fluctuating conditions in industrial-scale bioreactors.

For quantitative characterization of population distributions of single cell characteristics, mean fluorescence can be supplemented by calculation of peak width, mode and CV. A widening of distributions (higher peak width) combined with an increase in CV could be correlated to an increase in the level of population spread, respectively, increase in noise in gene expression. When correlating changes in these two parameters to mode and mean, which reveal information about fluorescence intensity, changes in the shape of a distribution can be suspected. Comparing mean and mode, mean is the better established parameter to describe averaged populations, while the mode only gives supplemental information when combined with the mean to evaluate potential skewness of the distribution. The appearance of shifts over time and differences in peak shape were quantified using cdf slopes and skewness, with higher slopes indicating narrower distributions and lower population heterogeneity. Also, the appearance of segregated subpopulations could be illustrated when the slope is significantly decreasing between consecutive samples. The skewness was able to monitor the appearance of subpopulations adjacent to the main population and additionally skews to both sides more sensitive than the slope. The reason might be bias during the determination of the exponential part of the cdf-plot. Furthermore, slope values are highly dependent on the setting of the flow cytometer e.g., using linear or log-scale. Therefore, we would recommend to use skewness instead of the slope of the cdf-plot, especially because population spreads can also be illustrated by the peak width.

When bi-modal subpopulations were visible as distinct subpopulations or adjacent to a main population, determining the percentage of the low- and high-fluorescence subpopulation could supplement and further increase the knowledge gained from quantitative analysis. However, it has to be mentioned that the analysis could still be extended to calculate the above-mentioned parameters for each of the subpopulations separately to e.g., evaluate the individual expression levels. Furthermore, if distributions that appeared as uni-modal hid further subpopulations our method is not able to uncover them. In such a case, deconvolution methods and algorithms already exist (e.g., Darzynkiewicz et al., 2017; Amalfitano et al., 2018; Corno and Callieri, 2018), and would need to be applied on the distribution data first, before calculation of the here introduced combination of parameters.

Generally, each parameter taken alone leads to an equal loss of information similar to just considering mean values, and therefore this study does not provide a “one-parameter”-solution, especially not when considering temporal changes of a series of distributions. However, combining mean, peak width or CV and skewness, that we identified as most descriptive parameters, enables quantitative description of distribution shape and fluorescence intensity. Of course, we did not develop a fully automated analysis tool including more advanced algorithms. However, the proposed analysis is easily applicable also by non-experts. Additionally, as indicated above, some parameters depend on the setting and alignment of the flow cytometer, but implementing quality control routines the results should be comparable for samples run on the same instrument. Nevertheless, these parameters generated with simple mathematical methods can be useful to among others process engineers during industrial process development and optimization aiming at for instance quantification of yield reduction caused by population heterogeneity or evaluation of physiological changes over time due to accumulation of a (toxic) by-product. Furthermore, these parameters might also be applied during early process development to screen for the most productive and robust subpopulation for a certain process goal by evaluating the temporal development of cell physiology or reaction to specific process conditions. During industrial manufacturing, these parameters could also contribute to process control strategies as based on single cell physiology e.g., addition of a feed could be controlled. So far this is to our knowledge only achieved based on average population level data. As shown in this study, the introduced combination of parameters is applicable for single cell studies of yeast as well as for *E. coli*, and can surely also be embedded easily in studies of other microorganisms and markers for population heterogeneity.

Considering the physiological findings, growth rate influenced the population distribution of membrane robustness for both yeast and *E. coli*. Slower-growing cells were largely unaffected by freeze-thaw treatment compared to rapidly growing cells and this effect was more pronounced for yeast than for *E. coli*. Freezing is well-known to affect and damage microbial cells. Thereby, cooling and thawing rates have a significant influence on the amount of damage inflicted (Mazur, 1984). Furthermore, consistent with our findings faster-growing cells are more susceptible to freeze-thaw treatment (Lewis et al., 1993), whereas slower-growing cells redistribute resources toward stress-tolerance functions (Dumont et al., 2004; Hua et al., 2004; Brauer et al., 2008; Zakrzewska et al., 2011). We previously analyzed the effects of freezing on fluorescence distributions and cell shape of the yeast reporter strain and observed no effects on cell morphology, but noted the formation of different GFP fluorescence subpopulations (Carlquist et al., 2012). In this study, we found that the underlying cause of the fluorescence decrease was mainly a pH drop as the extracellular and intracellular pH equalized via membrane pores in freeze-thaw treated yeast cells. Cell fluorescence was similar to unfrozen cells at external pH 7, and linearly decreased with decreasing external pH, consistent with the pH dependency of fluorescent proteins (Chudakov et al., 2010). These results verified that freezing reproducibly generated membrane pores that allowed ion but not fluorescent protein exchange between the intracellular and extracellular space, making freeze-thawing a feasible method for studying population heterogeneity in membrane robustness. The methodology was also successfully used to investigate heterogeneity in membrane robustness in *E. coli* cultures and might also be applicable for other microorganisms.

The influence of fluctuating environmental conditions that cells experience in industrial scale reactors was further explored by analyzing responses to transient changes from glucose limitation to glucose excess and back. While growth rate had only small effects on reporter gene expression in yeast and *E. coli*, glucose pulses resulted in clear reporter up-regulation, indicating that gene expression of the ribosomal promoters studied primarily responded to environmental shifts rather than growth rate at steady-state conditions. Slower-growing yeast and *E. coli* cells showed a greater increase in fluorescence intensity after a glucose pulse than faster-growing cells, indicating a noticeable shift in metabolism after relief of glucose limitation, as indicated by up-regulation of the reporter gene. Contrarily, membrane integrity was largely unchanged for slow-growing yeast and *E. coli* cultures after the glucose pulse. Fast-growing cultures of yeast and *E. coli* displayed different changes in population distributions in response to glucose pulses. *E. coli* cells showed an upshift in fluorescence mirroring growth phases during consumption of the pulsed glucose and an increased proportion of cells with intact membranes in the glucose-consumption phase. Also the yeast population distribution changed in response to glucose perturbation after freeze-thaw treatment. A large portion of the population in high-D cultures showed a dramatic decrease in membrane robustness. The reason why the glucose pulse seemed to protect the remaining portion of cells from freeze-thaw damage is unclear but must be related to intracellular metabolite levels because of the speed of the observed changes. We propose that the fast-growing cells were primed for overflow metabolism, quickly absorbed the extra glucose and might have produced storage carbohydrates such as trehalose, which accumulates in yeast in response to freeze stress (Mahmud et al., 2010). An intracellular shift in metabolite concentrations might affect the influence of freezing, protecting the cell membrane.

Nonetheless, high-throughput, single-cell gene expression studies are vital for elucidating the dynamics and contributions of heterogeneous cell populations. The combination of easy comprehensible parameters presented here combine reporter strains, flow cytometry and simple mathematical methods to objectively explore subpopulation dynamics in changing environments. The tools facilitate analysis and interpretation of flow cytometry data and provide insights for future strategies to improve the performance and robustness of industrial-scale cultivations.

## Data Availability

All data generated and analyzed during this study are included in this article and its supplementary material files. Raw datasets are available from the corresponding author on reasonable request.

## Author Contributions

A-LH designed and performed the experiments for the yeast part as well as the corresponding samples analysis, developed the method for data analysis, and did data interpretation and analysis for the yeast and *E. coli* part as well as drafted the manuscript. TJ and SH performed the experiments for the *E. coli* part and the corresponding sample analysis as well as revised the manuscript. LL assisted during sample analysis of both experimental parts and revised the manuscript. MC designed the experimental setup and supported the development of the data analysis method as well as revised the manuscript. KG, SS, and AE supported the development of the study concept, data interpretation, and revised the manuscript. All authors approved the final manuscript.

### Conflict of Interest Statement

TJ was employed by the company Glycom A/S. The remaining authors declare that the research was conducted in the absence of any commercial or financial relationships that could be construed as a potential conflict of interest.
