# Supplementary material for: Quantitative Flow Cytometry to Understand Population Heterogeneity in Response to Changes in Substrate Availability in Escherichia coli and Saccharomyces cerevisiae Chemostats
Source: Front Bioeng Biotechnol. 2019 Aug 5;7:187. doi: 10.3389/fbioe.2019.00187 (PMC6691397; doi:10.3389/fbioe.2019.00187)
Supplement: Supplementary file 1 [file Data_Sheet_1.docx]

***Supplementary Material***

**S1: Physiological characteristics of *S. cerevisiae* steady-state cultures**

*S. cerevisiae* steady-state cultures were characterized by the yields of cell mass, CO_2_, ethanol and acetate on glucose, as well as the uptake respectively production rate of glucose and ethanol (Table S1). Carbon balances were calculated from yield coefficients to confirm data consistency. For D = 0.05 h^-1^, the yield of biomass (Y_SX_), was around 58% (Table S1), consistent with literature data for respiratory metabolism (Dijken et al., 2000). No metabolite accumulation was detected, indicating absence of overflow metabolism, which is in accordance with earlier observations (Postma et al., 1989). For D = 0.3 h^- 1^, yield of biomass was lower and accompanied by accumulation of ethanol and small amounts of acetate as also seen in (Diderich et al., 1999). These results were in agreement with previous results showing the onset of respiro-fermentative metabolism at D = 0.28 h^-1^ (Hoek et al., 1998) for the laboratory strain CEN.PK113-7D upon which our strain is based. Yield of CO_2_ (Y_SCO2_) decreased with increasing D, whereas specific substrate uptake rate (r_S_) and specific ethanol production rate (r_Ethanol_) increased in agreement with (Postma et al., 1989) and (Hoek et al., 1998). All yields showed low standard deviations confirming steady-state on physiological level and high reproducibility between replicates. Carbon balances closed within 8% for the highest D and 11% for the lowest D. This carbon balance variation could be explained by differences in biomass yield compared to earlier studies and absence of glycerol production (Larsson et al., 1993; Hoek et al., 1998).

**Table S**1 **yields, carbon balances and consumption respectively production rates for glucose and ethanol for aerobic, glucose-limited continuous cultures of *S. cerevisiae* FE440 at different dilution rates.**

| **Chemostat parameter** | **D=0.05 h^-1^** | **D=0.3 h^-1^** |
| --- | --- | --- |
| **Y_SX_** _[C-mole/C-mole]_ | 0.58±2·10^-2^ | 0.22± 1·10^-2^ |
| **Y_SCO2_** _[C-mole/C-mole]_ | 0.53± 2·10^-2^ | 0.27± 1·10^-2^ |
| **Y_SEth_** _[C-mole/C-mole]_ | - | 0.42± 1·10^-2^ |
| **Y_SAce_** _[C-mole/C-mole]_ | - | 0.01± 1·10^-4^ |
| **r_S_** _[gGlucose/gCells·h]_ | 0.10± 1·10^-2^ | 1.61± 5·10^-2^ |
| **r_Ethanol_** _[gEthanol/gCells·h]_ | - | 0.52± 1·10^-4^ |
| **Carbon balance** | **1.11± 5·10^-2^** | **0.92± 1·10^-2^** |

D, dilution rate; Y_SX_, growth yield on glucose; Y_SCO2_, yield of CO_2_ on glucose; Y_SEth_, yield of ethanol on glucose; Y_SAce_, yield of acetate on glucose; r_S_, specific glucose uptake rate; r_Ethanol_, specific ethanol production rate; Yields are ratios of cell mass, CO_2_, ethanol respectively acetate per amount of consumed substrate. Data are presented with standard deviations obtained from biological triplicates from samples taken at three subsequent residence times.

**S2: Amphotericin Test *S. cerevisiae***

The robustness of bioreactor populations was tested using the *S.* *cerevisiae* reporter strain to identify subpopulations with different fluorescence responses to freeze-thaw stress, with the GFP signal as a measure of membrane integrity (Carlquist et al., 2012). The main underlying reason for reduced cellular fluorescence after freezing was suspected to be a reduction in GFP fluorescence originating from a shift towards lower intracellular pH due to leaking membranes and the low extracellular pH, which was kept at 5. However, actual loss of intracellular GFP may also be a cause. To evaluate the mechanism further, yeast cells were permeabilised with Amphotericin B followed by incubation in buffers with different pH before flow cytometry analysis. Amphotericin B is an antifungal drug, which binds [ergosterol](http://en.wikipedia.org/wiki/Ergosterol) in membranes, forming transmembrane pores that allow the passage of ions but not proteins (Medoff et al., 1972). Flow cytometry analysis of the permeabilised yeast cells exhibited a linear decrease in fluorescence as the external pH dropped below 7 (data not shown). As the values obtained for incubations with a buffer at pH 5 were equal to those seen for frozen cells, it was concluded that the reduction in fluorescence caused by freezing is dominated by changes in intracellular pH.

**S3: physiological response of *S. cerevisiae* to glucose perturbation**

Cultures with a low dilution rate of D= 0.05 h^-1^ (Figure S1) displayed batch-culture-like behavior with sharp increase in CO_2_ production, overflow metabolism and concomitant ethanol and acetate accumulation when perturbed with a 1 g.L^-1^ glucose pulse, consistent with (Visser et al., 2004). In contrast, respiro-fermentative cultures (D= 0.3 h^-1^) showed slight increases in CO_2_ generation, consumed the extra glucose at a slower rate than slower-growing cultures and did not show increased ethanol or acetate production.


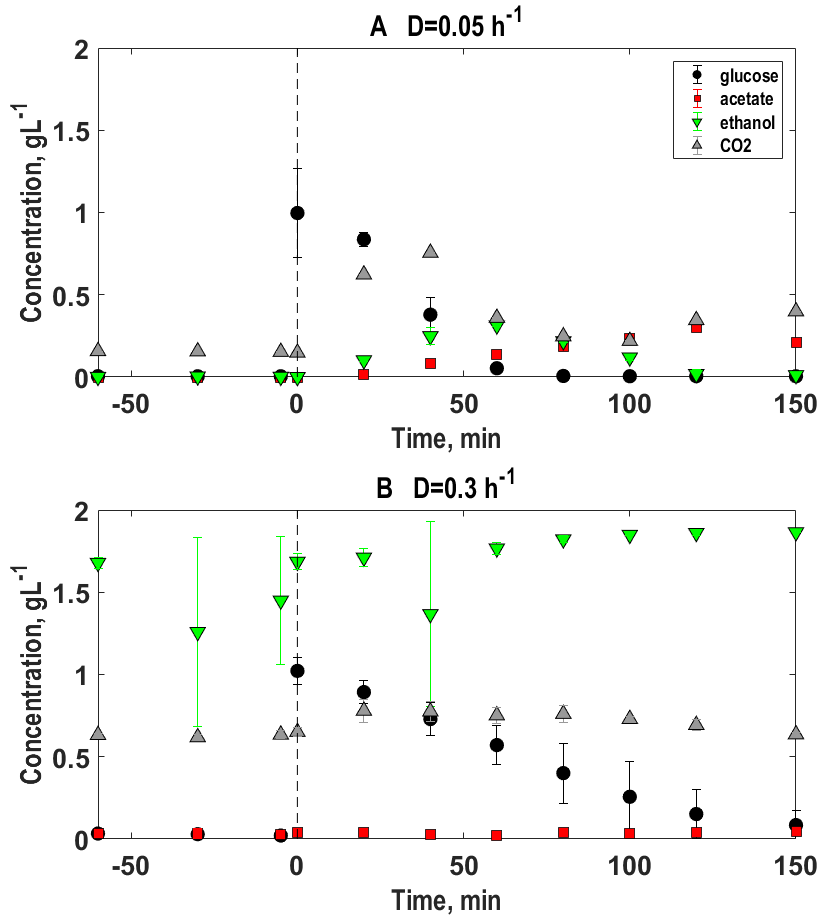


**Fig. S1 yeast culture responses to glucose pulse.** A 1 g.L^-1^ glucose pulse was introduced to aerobic, glucose-limited chemostats of *S. cerevisiae* growing at D = 0.05 h^-1^ (top) and 0.3 h^-1^ (bottom). Glucose (g.L^- 1^, black), CO_2_ (v/v %, grey), ethanol (g.L^-1^, green) and acetate (g.L^-1^, red) concentrations are shown before and after the glucose pulse including error bars for biological triplicates. Dashed lines mark the time point of the pulse

**S4: *E. coli* freezing test**

Prior to population heterogeneity experiments using the *E. coli* GFP-reporter strain to measure membrane robustness after freeze-thaw stress, experiments were conducted on fresh and frozen cells to determine if freezing resulted in leaky membranes that could be detected as a reduction in GFP signal in cells kept in buffers at pH 5.5–7.5.

**Materials and Methods.** Freezing tests were done in shaker flasks using minimal medium (Xu et al., 1999) as in chemostat cultures. Triplicate samples were collected from each growth phase and divided into three subsamples for direct measurement, flash-freezing in liquid nitrogen with 15 % glycerol, and slow-freezing at -80 °C with 15% glycerol. To avoid degradation, 20 samples were thawed at a time, centrifuged, and re-suspended in 0.9% NaCl. The maximum lag between the first and tenth analyzed samples was 10 min. Re-analysis of first samples after analysis of last samples gave identical results, indicating that intracellular GFP concentration was not influenced by sample preparation (data not shown).

**Results.** Frozen cells analyzed at pH 7 displayed fluorescence pattern similar to fresh cells, independent of growth phase or freezing method (data not shown). Fluorescence distribution remained the same with and without freezing for *E. coli* cells analyzed at pH 6.5–7.5 (Figure S2). In contrast, GFP fluorescence of freeze-thawed cells declined when the cells were re-suspended in buffers below pH 6 consistent with yeast results and the pH dependence of GFP (Robey et al., 1998). Based on these results, we analyzed the membrane robustness of *E. coli* using GFP fluorescence measurements of frozen cells at pH 5.5 and 7, with GFP fluorescence at pH 7 as reference for intact cells.

**
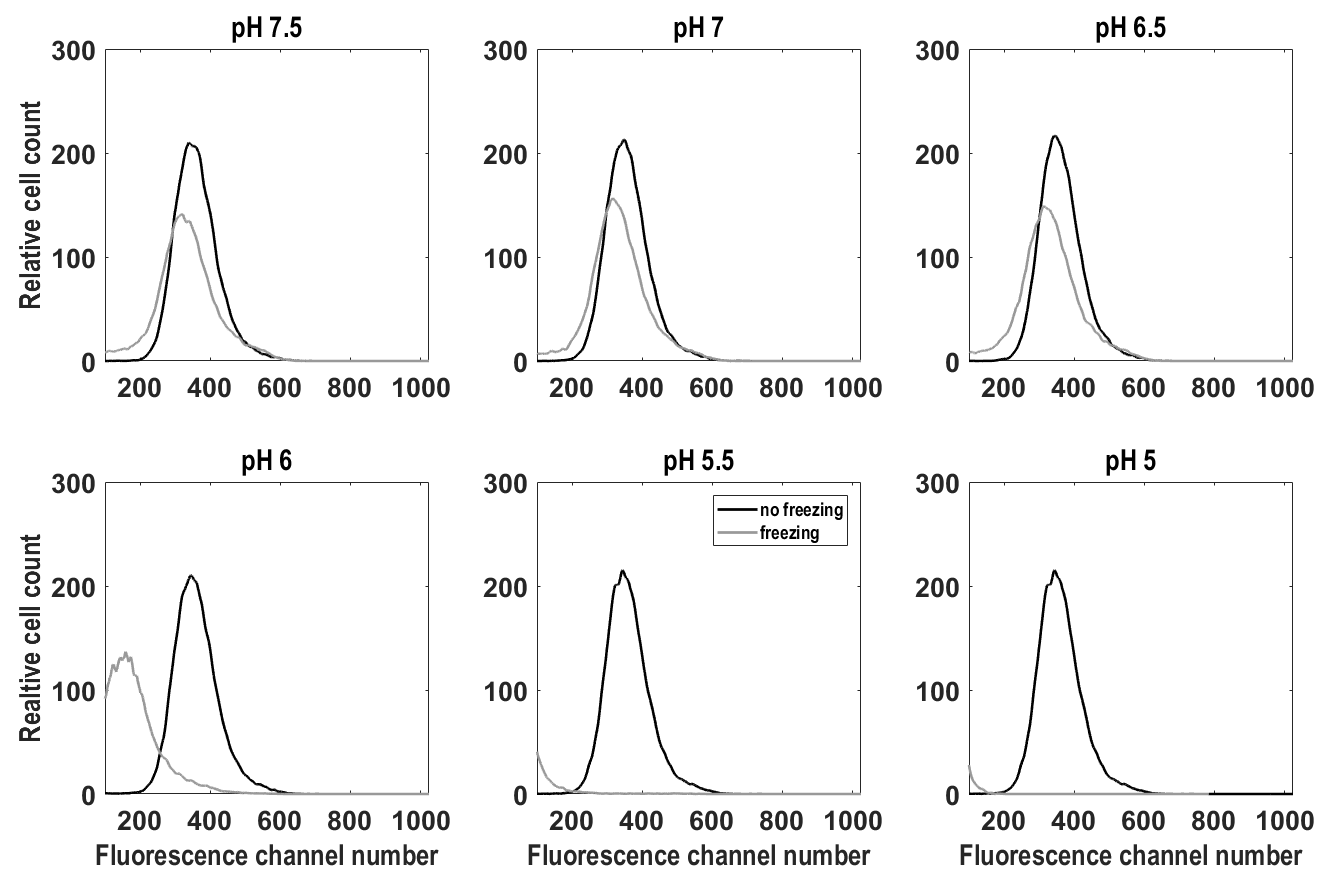
**

**Fig. S2 effect of freeze-thaw stress on GFP signal distribution of *E. coli* cells at different pH values.** Histograms for GFP fluorescence of the *E. coli* reporter strain analyzed at pH 5.5–7.5. Black, non-frozen cells; grey, frozen cells

**S5: Physiology of *E. coli* cells at steady-state conditions**

Yields on glucose for cell mass, CO_2_ and metabolites produced in significant amounts, as well as the computed glucose uptake were determined for steady-state *E. coli* cultures (Table S2). Carbon balances were calculated from yield coefficients to confirm data consistency. Biomass yields on glucose were 0.44 cmole/cmole for the low D and 0.57 cmole/cmole for high D. Yields were lower than reported in literature for *E. coli* K12 on the same medium: biomass yields of 0.6 cmole/cmole are reported for dilution rates below 0.4 h^-1^ and biomass yield increases with increasing D to a maximum of 0.69 cmole/cmole before tapering at higher rates (Kayser et al., 2005). Similar to *S. cerevisiae* cultures, at low growth rates (D = 0.1 h^-1^ and D = 0.36 h^-1^), *E. coli* cultures showed strict respiratory metabolism with no acetate formation. Literature data suggest a shift to respiro-fermentative growth at D values above 0.4 h^-1^ with concomitant acetate production (Nanchen et al., 2006). However, we observed only a small amount of acetate at the highest growth rate of D = 0.51 h^-1^, consistent with (Hua et al., 2004), who detected no acetate excretion with biomass yields that were similar to our results. We also observed low lactate concentrations (<0.1 g.L^-1^) (data not shown), but formation of formate at higher D values. Surprisingly, also small amounts of glycerol were produced at all tested D values. Our low standard deviations for calculated yields and carbon balances closing with a maximum error of 5% demonstrated high data consistency.

**Table S2 yields, carbon balance and glucose consumption rate at different dilution rates for aerobic, glucose-limited *E. coli* MG1655/pGS20PfisGFPAAV chemostat cultures**.

| **Chemostat parameter** | **D =0.1 h^-1^** | **D =0.36 h^-1^** | **D =0.51 h^-1^** |
| --- | --- | --- | --- |
| **Y_SX_** _[C-mole/C-mole]_ | 0.44± 2·10^-2^ | 0.58±2·10^-2^ | 0.56± 2·10^-2^ |
| **Y_SCO2_** _[C-mole/C-mole]_ | 0.49± 1·10^-4^ | 0.37± 1·10^-4^ | 0.36± 1·10^-4^ |
| **Y_SAce_** _[C-mole/C-mole]_ | 0.001± 1·10^-4^ | 0.001± 5·10^-4^ | 0.003± 1·10^-3^ |
| **Y_SGly_** _[C-mole/C-mole]_ | 0.007± 2·10^-3^ | 0.003± 1·10^-4^ | 0.004± 1·10^-3^ |
| **Y_SFor_** _[C-mole/C-mole]_ | - | 0.03± 3·10^-3^ | 0.03± 1·10^-3^ |
| **r_S_** _[gGlucose/gCells·h]_ | 0.18± 9·10^-2^ | 0.78± 4·10^-2^ | 0.94± 1·10^-4^ |
| **Carbon balance** | **0.95± 2·10^-2^** | **0.98± 2·10^-2^** | **0.96± 2·10^-2^** |

D, dilution rate; Y_SX_, growth yield on glucose; Y_SCO2_, yield of CO_2_ on glucose; Y_SAce_, yield of acetate on glucose; Y_SGly_, yield of glycerol on glucose; Y_SFor_, yield of formate on glucose; r_S_, specific glucose uptake rate; Yields are ratios of cell mass, CO_2_, acetate, glycerol respectively formate per amount of consumed substrate. Data are presented with standard deviations obtained from biological triplicates with samples taken at three subsequent residence times.

**S6: physiological response of *E. coli* to glucose perturbation**

Pulsed glucose was depleted after 30 min for D = 0.1 h^-1^ and after 37 min for D = 0.36 h^-1^ and D = 0.51 h^- 1^ (Figure S3). No formation of acetate or other metabolites was detected as a consequence of the pulse at 0.1 h^- 1^ consistent with earlier findings for respiratory metabolism (Kayser et al., 2005). Due to overflow metabolism, small amounts of acetate and glycerol were present at steady-state for D = 0.36 h^-1^ and D = 0.51 h^-1^ and increased up to 0.1 g.L^-1^ respectively 0.15 g.L^-1^ after the pulse (connected with a rise in CO_2_). After glucose depletion, acetate was consumed connected to a second rise in CO_2_ level. Acetate reduction was slower for D = 0.51 h^-1^ than for D = 0.36 h^-1^, because cells were already in overflow metabolism and unable to re-assimilate produced acetate, which was probably diluted rather than consumed.


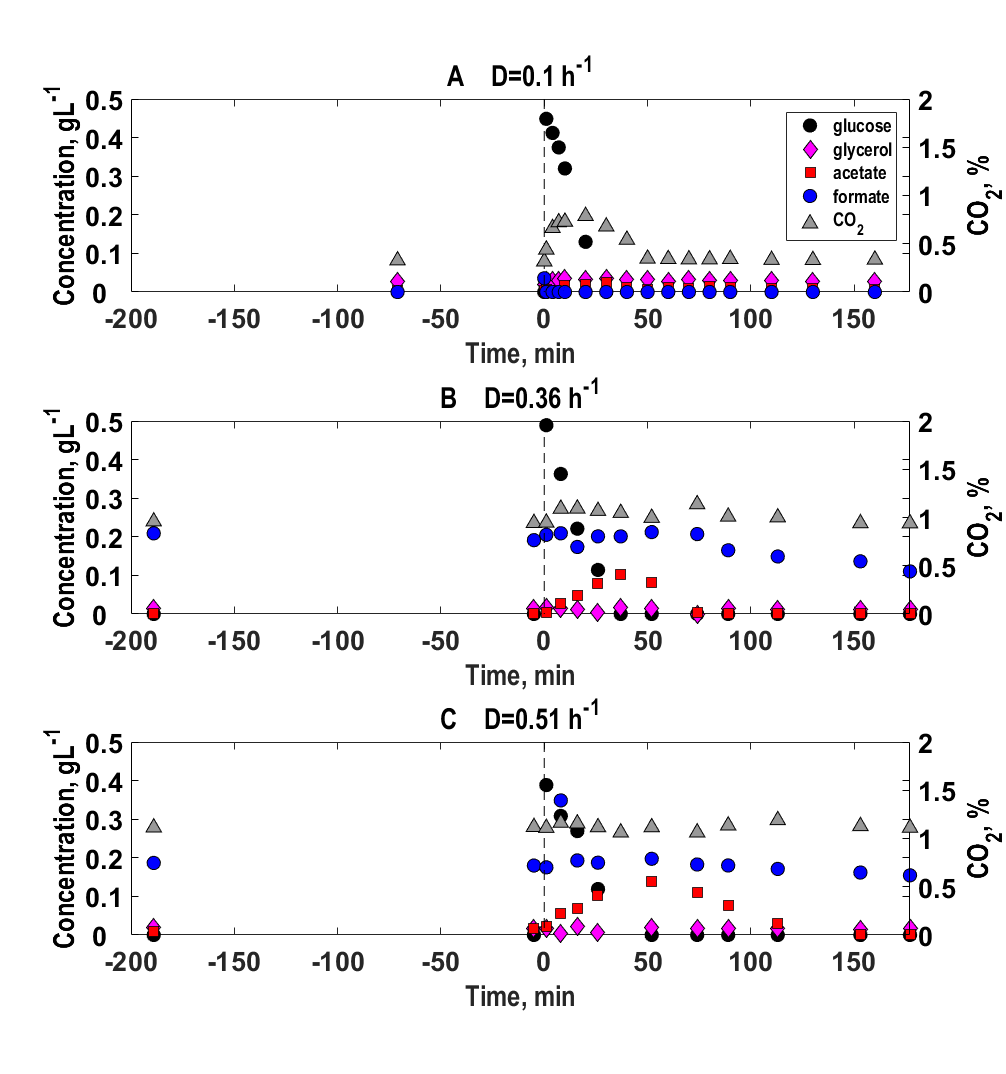


**Fig. S3** **perturbation of *E. coli* cultures after 0.45 g.L^-1^ glucose pulse.** Shown are profiles for aerobic, glucose-limited chemostat cultures grown at D = 0.1 h^-1^ (A), 0.36 h^-1^ (B), 0.51 h^-1^ (C). Glucose (g.L^-1^, black), acetate (g.L^-1^, red), glycerol (g.L^-1^, pink), CO_2_ (v/v%, grey) and formate (g.L^-1^, blue) concentrations before and after the glucose pulse including error bars for biological triplicates

**References**

Carlquist, M., Lencastre Fernandes, R., Helmark, S., Heins, A.L., Lundin, L., Sorensen, S.J., et al. (2012). Physiological heterogeneities in microbial populations and implications for physical stress tolerance. *Microbial cell factories* 11(94).

Diderich, J.A., Schepper, M., Hoek, P., Luttik, M.A.H., Dijkens, J.P., Pronk, J.T., et al. (1999). Glucose Uptake Kinetics and Transcription of HXT Genes in Chemostat Cultures of *Saccharomyces cerevisiae.* *Journal of biological chemistry* 274(22)**,** 15350-15359.

Dijken, J.P., Bauer, J., Brambilla, L., Duboc, P., Francois, J.M., Gancedo, C., et al. (2000). An interlaboratory comparison of physiological and genetic properties of four *Saccharomyces cerevisiae* strains. *Enzyme and Microbial Technology* 26**,** 706-714.

Hoek, P., Dijken, J.P., and Pronk, J.T. (1998). Effect of Specific Growth Rate on Fermentative Capacity of Baker’s Yeast. *Applied and Environmental Microbiology* 64(11)**,** 4226-4233.

Hua, Q., Yang, C., Oshima, T., Mori, H., and Shimizu, K. (2004). Analysis of Gene Expression in *Escherichia coli* in Response to Changes of Growth-Limiting Nutrient in Chemostat Cultures. *Applied and Environmental Microbiology* 70(4)**,** 2354-2366. doi: 10.1128/aem.70.4.2354-2366.2004.

Kayser, A., Weber, J., Hecht, V., and Rinas, U. (2005). Metabolic flux analysis of *Escherichia coli* in glucose-limited continuous culture. I. Growth-ratedependent metabolic efficiency at steady state. *Microbiology-Sgm* 151**,** 693-706. doi: Doi 10.1099/Mic.0.27481-0.

Larsson, C., Stockar, U., Marison, I., and Gustafsson, L. (1993). Growth and Metabolism of *Saccharomyces cerevisiae* in Chemostat Cultures under Carbon-, Nitrogen-, or Carbon- and Nitrogen-Limiting Conditions. *Journal of bacteriology* 175(15)**,** 4809-4816.

Medoff, G., Kobasyashi, G.S., Kwan, C.N., Schlessinger, D., and Venkov, P. (1972). Potentiation of Rifampicin and 5-Fluorocystosine as antifungal antibiotics by Ampothericin B. *PNAS* 69(1)**,** 196-199.

Nanchen, A., Schicker, A., and Sauer, U. (2006). Nonlinear dependency of intracellular fluxes on growth rate in miniaturized continuous cultures of *Escherichia coli.* *Appl Environ Microbiol* 72(2)**,** 1164-1172. doi: 10.1128/AEM.72.2.1164-1172.2006.

Postma, E., Verduyn, C., Scheffers, W.A., and Dijken, J.P. (1989). Enzymic Analysis of the Crabtree Effect in Glucose-Limited Chemostat Cultures of *Saccharomyces cerevisiae.* *Applied and Environmental Microbiology* 55(2)**,** 468-477.

Robey, R.B., Ruiz, O., Santos, A.V.P., Ma, J., Kear, F., Wang, L.-J., et al. (1998). pH-Dependent Fluorescence of a Heterologously Expressed Aequorea Green Fluorescent Protein Mutant: In Situ Spectral Characteristics and Applicability to Intracellular pH Estimation. *Biochemistry* 37**,** 9894-9901.

Visser, D., van Zuylen, G.A., van Dam, J.C., Eman, M.R., Proll, A., Ras, C., et al. (2004). Analysis of in vivo kinetics of glycolysis in aerobic *Saccharomyces cerevisiae* by application of glucose and ethanol pulses. *Biotechnol Bioeng* 88(2)**,** 157-167. doi: 10.1002/bit.20235.

Xu, B., Jahic, M., Blomsten, G., and Enfors, S.O. (1999). Glucose overflow metabolism and mixed-acid fermentation in aerobic large-scale fed-batch processes with *Escherichia coli*. *Appl Microbiol Biotechnol* (51)**,** 564-571.
